# Supplementary material for: Dynamic transcriptomic profiles of zebrafish gills in response to zinc supplementation
Source: BMC Genomics. 2010 Oct 11;11:553. doi: 10.1186/1471-2164-11-553 (PMC3091702; doi:10.1186/1471-2164-11-553)
Supplement: Additional file 2 — Interactive Direct Interaction Network representing the molecular interactions between zinc, copper, iron, calcium and proteins encoded by transcripts changed by zinc supplementation. Mini web-site containing index.html and hyperlinked pages in subdirectory describing a Direct Interaction Network automatically generated based on curated interactions contained within the proprietary PathwayArchitect database. Ovals represent proteins and the circles symbolize metal ions. Objects are coloured by their abundance in zebrafish at the time-point they were significantly different from the control is a scale from -4 fold (dark green) to +4 fold (dark red). Where significant differences were found at more than one time-point, the colour overlay shows expression at the first instance. Dark blue squares denote 'binding', and light blue squares 'expression'; green squares stand for 'regulation', green diamonds for 'metabolism', and green circles for 'promoter binding'. Arrow heads indicate directionality of the interaction where annotated. All nodes and edges can be further interrogated by selecting the relative area of the image. [file 1471-2164-11-553-S2.zip › PathwayArchitect Zn xs DIN/84530.html]

# PROTEIN: NDRG1

|  |  |
| --- | --- |
| Name | NDRG1 |
| Type | PROTEIN |
| Description | N-myc downstream regulated gene 1 |
| Note | This gene is a member of the N-myc downregulated gene family which belongs to the alpha/beta hydrolase superfamily. The protein encoded by this gene is a cytoplasmic protein involved in stress responses, hormone responses, cell growth, and differentiation. Mutation in this gene has been reported to be causative for hereditary motor and sensory neuropathy-Lom. |
| Alias | CMT4D |
|  | GC4 |
|  | nickel-specific induction protein Cap43 |
|  | protein regulated by oxygen-1 |
|  | N-myc downstream regulated 1 |
|  | Ndr1 |
|  | tunicamycin-responsive protein |
|  | NMSL |
|  | PROXY1 |
|  | RTP |
|  | RIT42 |
|  | NDR1 |
|  | differentiation-related gene 1 protein |
|  | TDD5 |
|  | TARG1 |
|  | CAP43 |
|  | N-myc downstream regulated gene 1 protein |
|  | reducing agents and tunicamycin-responsive protein |
|  | HMSNL |
|  | DRG1 |


---

|  |  |
| --- | --- |
| GO Component | nucleus |


---

|  |  |
| --- | --- |
| GO ID | GO:0005634 |
|  | GO:0030154 |
|  | GO:0045576 |
|  | GO:0003824 |
|  | GO:0010038 |


---

|  |  |
| --- | --- |
| MIM | MIM:601455 |
|  | MIM:605262 |


---

|  |  |
| --- | --- |
| Connectivity | 127 |


---

|  |  |
| --- | --- |
| Entrez ID | 17988 |
|  | 10397 |


---

|  |  |
| --- | --- |
| Agilent ID | A\_53\_P169391 |
|  | A\_14\_P100016 |
|  | A\_23\_P20494 |
|  | A\_14\_P107381 |
|  | A\_53\_P140014 |
|  | A\_24\_P38387 |
|  | A\_14\_P201418 |
|  | A\_14\_P129210 |
|  | A\_51\_P405606 |
|  | A\_53\_P136761 |
|  | A\_53\_P171392 |
|  | A\_53\_P136020 |
|  | A\_14\_P111630 |
|  | A\_53\_P139084 |


---

|  |  |
| --- | --- |
| Cellular Localization | Nucleus |
|  | Organelle |
|  | Cell |


---

|  |  |
| --- | --- |
| Pathway | Zn xs inventory |
|  | Zn xs DIN |


---

|  |  |
| --- | --- |
| GO Process | cell differentiation |
|  | mast cell activation |
|  | response to metal ion |


---

|  |  |
| --- | --- |
| UniGene | Mm.30837 |
|  | Hs.372914 |


---

|  |  |
| --- | --- |
| Affymetrix Probeset ID | 240991\_at |
|  | 1423413\_at |
|  | 1450976\_at |
|  | 1456174\_x\_at |
|  | 200632\_s\_at |
|  | 1450977\_s\_at |
|  | 36933\_at |
|  | 78954\_at |
|  | D87953\_at |
|  | g5174656\_3p\_s\_at |
|  | 99125\_at |
|  | Hs.271827.0.A1\_3p\_at |
|  | 96596\_at |
|  | U52073\_s\_at |


---

|  |  |
| --- | --- |
| GO Function | catalytic activity |


---

|  |  |
| --- | --- |
| Nucleotide | CR456842 |
|  | AK096562 |
|  | BC006260 |
|  | AK199494 |
|  | AU141826 |
|  | D87953 |
|  | BX648361 |
|  | AK223542 |
|  | AK098075 |
|  | AY277597 |
|  | AK124709 |
|  | BC003175 |
|  | AK161860 |
|  | AF039944 |
|  | AK170281 |
|  | AF004162 |
|  | X92845 |
|  | AK129867 |
|  | AK165007 |
|  | BC071235 |
|  | AK170809 |
|  | AF147402 |
|  | AK091269 |
|  | AK126924 |
|  | BC015282 |
|  | AK194260 |
|  | U60593 |
|  | NM\_006096 |
|  | AK004548 |
|  | AK170636 |
|  | AK188581 |
|  | AK095649 |
|  | AK200211 |
|  | AB004248 |
|  | AK170439 |
|  | AF230380 |
|  | NM\_010884 |
|  | AK091147 |


---

|  |  |
| --- | --- |
| Protein | AAQ18036 |
|  | Q92597 |
|  | AAF71305 |
|  | CAA63430 |
|  | AAC13419 |
|  | AAH71235 |
|  | BAD97262 |
|  | BAE36611 |
|  | BAC04597 |
|  | BAE41927 |
|  | AAH15282 |
|  | Q62433 |
|  | BAE42042 |
|  | NP\_035014 |
|  | BAA13505 |
|  | AAH03175 |
|  | BAE41797 |
|  | BAE41683 |
|  | CAG33123 |
|  | NP\_006087 |
|  | AAB03484 |
|  | BAB23362 |
|  | AAH06260 |


---

|  |  |
| --- | --- |
| Organism | Mammal |


---

|  |  |
| --- | --- |
| Location | chromosome 8, 8q24.3 (Homo sapiens) |
|  | chromosome 15, 15 D2 (Mus musculus) |


---

|  |  |
| --- | --- |
